# The levels of serum lipoprotein(a) on clinical outcomes in Chinese hospitalized patients with cardiovascular diseases

**DOI:** 10.1186/s40001-024-01957-7

**Published:** 2024-08-17

**Authors:** Tingting Min, Yilin Yue, Xin Fan, Deguang Yang, Shaohui Su, Huaibin Wan

**Affiliations:** 1 Department of Cardiology, Dongguan People’s Hospital, The First School of Clinical Medicine, Southern Medical University, Dongguan, 523059 Guangdong People’s Republic of China; 2 Department of Cardiology, Shenhe People’s Hospital, The Fifth Affiliated Hospital of Jinan University, Heyuan, 517000 Guangdong People’s Republic of China

**Keywords:** Lipoprotein(a), Outcomes, Cardiovascular diseases, Retrospective study

## Abstract

**Objective:**

Serum lipoprotein(a) [Lp(a)] is a risk factor of cardiovascular diseases. However, the relationship between the serum Lp(a) and clinical outcomes has been seldom studied in Chinese hospitalized patients with cardiovascular diseases.

**Methods:**

We retrospectively collected the clinical data of hospitalized patients with cardiovascular diseases in the Cardiovascular Department of Dongguan People’s Hospital from 2016 to 2021 through the electronic case system. Patients were divided into 4 groups based on Lp(a) quartiles: Quartile1 (≤ 80.00 mg/L), Quartile 2 (80.01 ~ 160.90 mg/L), Quartile 3 (160.91 ~ 336.41 mg/L), Quartile 4 (> 336.41 mg/L). Cox proportional hazard regression models were constructed to examine the relationship between Lp(a) and cardiovascular events.

**Results:**

A total of 8382 patients were included in this study. After an average follow-up of 619 (320 to 1061) days, 1361 (16.2%) patients developed major adverse cardiovascular events, and 125 (1.5%) all-cause death were collected. The incidence of MACEs was 7.65, 8.24, 9.73 and 10.75 per 100 person-years in each Lp(a) quartile, respectively; the all-cause mortality was 0.48, 0.69, 0.64 and 1.18 per 100 person-years in each Lp(a) quartile, respectively. The multivariate Cox regression analysis suggested that high Lp(a) level was an independent risk factor for MACEs (HR: 1.189, [95% CI: 1.045 to 1.353], *P* = 0.030) and all-cause death (HR: 1.573, [95% CI: 1.009 to 2.452], *P* = 0.046).

**Conclusion:**

In addition to traditional lipid indicators, higher Lp(a) exhibited higher risks of adverse cardiovascular events and death, indicated worse prognosis. Lp(a) may be a new target for the prevention of atherosclerotic diseases.

## Introduction

Lipoprotein(a) [Lp(a)] has been first proposed about 60 years ago [1]. As a component of blood lipids, Lp(a) levels are generally not influenced by lifestyle [2]. Early in 1986, Dahlen et al. [3] had found that Lp(a) was shown to be an independent predictor of the presence of coronary lesions, and might be more potent in promoting atherosclerosis than low density lipoprotein (LDL). After this, many studies had established a strong link between Lp(a) and cardiovascular diseases [4–6]. Pathology and genetics indicated that evaluated Lp(a) levels can increase the risk of cardiovascular diseases by causing atherosclerosis and thrombosis [7]. First, Lp(a) can decrease the generation of plasmin, and promote a thrombosis in arteries. Because apolipoprotein-A (apo-A), as a component of Lp(a), contains repeated kringle (K) structures (KIV and KV), Lp(a) is highly homologous to plasminogen [8]. Ishikawa et al. found that low serum Lp(a) could increase the risk of cerebral hemorrhage [9], which indicated that Lp(a) might be related to coagulation. Secondly, high concentrations of Lp(a) can result in accumulation of Lp(a) into the arterial wall [10] and promote atherosclerosis. In the process, apo-A plays a key role in anchoring Lp(a) to the extracellular matrix within the arteries [11, 12]. Meanwhile, Lp(a) could increase the risk of suffering from valvular aortic diseases (VAS), by promoting cholesterol deposition in the arterial intima and aortic valve leaflets [13]. To our knowledge, few studies paid concentration to the relationship between serum Lp(a) levels and clinical outcomes in Chinese patients. In this study, we retrospectively explored the relationship between serum Lp(a) concentrations and the incidence of adverse events in Chinese hospitalized patients with cardiovascular diseases.

## Methods

### Study population

This retrospective study was designed for exploring the link between risk factors and clinical outcomes in hospitalized patients with cardiovascular diseases. Clinical data, visits, and laboratory results of 15,210 patients admitted to the Department of Cardiology from 2016 to 2021 were retrieved from Dongguan People’s Hospital. The inclusion criteria were: patients diagnosed with cardiovascular-related diseases based on the International Statistical Classification of Diseases and Related Health Problems 10th Revision, including: I05-15, I20-25, I34-36, I42, and I44-50. The exclusion criteria were as follows: (1) age < 18 years old or > 75 years old; (2) patients who did not agree to follow-up or attend any clinical studies; (3) patients who were missed the data of medical record data, laboratory results and medicine; (4) patients with expected survival < 1 month. The endpoints were set at the final date of the follow-up. This study is exempt from the need for informed consent according to China’s “Ethical Review Approaches for Biomedical Research Involving Humans” 2016, Article 39[14]. And the study was approved by the institute ethics committee and was conducted following the Declaration of Helsinki (Ethics Approval Number: KYKT2022-012).

### Data collection

Age, sex, residence information, smoking habit and first blood biochemical results (including serum low density lipoprotein cholesterol (LDL-c), high density lipoprotein cholesterol (HDL-c), total cholesterol (TC), triglyceride (TG) and Lp(a) were collected through the Goodwill medical record system (Goodwill, CN). The patients’ primary diagnosis at discharge were used as the baseline medical history, and the major comorbidities such as diabetes, stroke and chronic kidney disease (CKD) were recorded. The use of statins, aspirin, clopidogrel, angiotensin-converting enzyme inhibitors (ACEIs), angiotensin receptor blockers (ARBs), β-blockers and calcium channel blockers (CCBs) at discharge were collected. The patients’ comorbidities were collected and calculated Charlson Comorbidity Index (CCI) [15]. The collection of laboratory results were limited to fasting blood samples within 24 h after admission according to the clinical laboratory requirements. Lp(a), LDL-c, TC, HDL-c and TG were measured using clinical laboratory methods (Beckman CX9, USA).

### Follow-up and outcomes

The expected follow-up time was 3 years, and the follow-up would be stopped if patients developed primary endpoint events. The definition of primary endpoint was composite of major adverse cardiovascular events (MACEs), including all-cause death, non-fatal myocardial infarction, non-fatal stroke, unstable angina, heart failure. And the secondary endpoint was all-cause death. An independent research team evaluated patients’ status and endpoint events according to the follow-up records in medical record system. Any inconsistencies were resolved by the researchers through telephone interviews with the treating physician and/or patients.

### Statistical analysis

All patients were divided into 4 groups based on serum Lp(a) concentrations quartiles: Quartile1 (≤ 80.00 mg/L), Quartile 2 (80.01 ~ 160.90 mg/L), Quartile 3 (160.91 ~ 336.41 mg/L), Quartile 4 (> 336.41 mg/L). The continuous variables were expressed as mean ± standard deviation (SD) or median and inter-quartile range (IQR), and tested by ANOVA or nonparametric test, respectively. The categorical variables were expressed as frequencies (n) and percentages (%), and the differences between groups were evaluated by Pearson Chi-square test. Patients who were lost to follow-up were continued to be included in outcome analysis. Patients’ survival in different groups were described by Kaplan–Meier analysis and log-rank test. Cox proportional hazard regression models were constructed to examine the relationship between Lp(a) and endpoint, and the Schoenfeld residuals were used to test the proportional hazard assumption of Cox model. In order to avoid the influence of overfitting, the variables which were closely correlated with MACEs and all-cause death for COX regression analysis were selected. The principles include the following two points: (1) there is a significant statistical difference in statistical analysis (*P* < 0.10); (2) previous studies have shown a correlation between variables and analysis events. The area under curve (AUC) of receiver operating characteristic (ROC) analysis were used to evaluate the diagnostic value of high Lp(a) levels and other biomarkers for cardiovascular adverse events. A *P*-value < 0.05 was considered to be statistically significant. Statistical analysis was performed using SPSS 22.0 software package for Windows (IBM, USA).

## Results

**Fig. 1:**
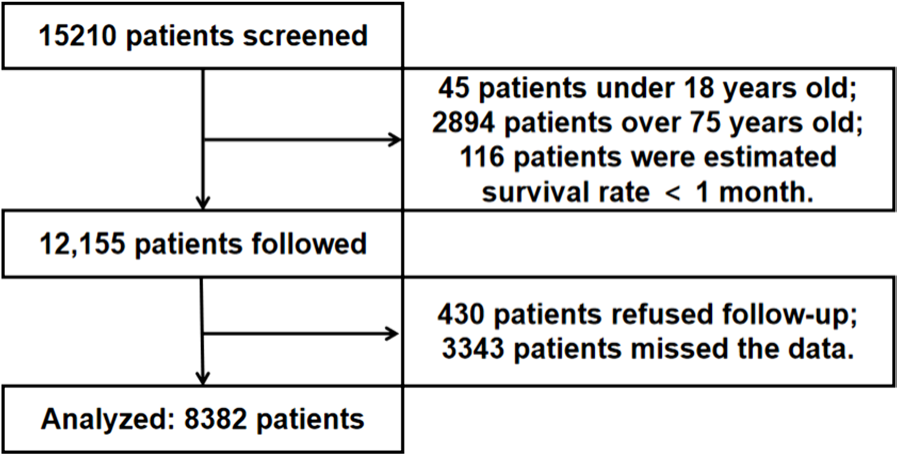
A flow diagram of the study

A total of 8382 patients were analyzed (as shown in Fig. 1). The mean age of the patients was 61 (51 to 68) years old; 5348 (63.8%) were male; 6504 (77.6%) were local residents; 3060 (36.5%) had a smoking habit. And there were 4338 (51.8%) patients with coronary heart diseases (CHDs); 3048 (36.4%) patients with acute coronary syndrome (ACS); 5117 (61.0%) patients with hypertension; 976 (11.6%) patients with CKD, and 1117 (13.3%) patients with stroke.

As is shown in Table 1, the baseline LDL-c concentration was 3.16 (2.53 to 3.82) mmol/L, HDL-c concentration was 1.09 (0.93 to 1.30) mmol/L, TC concentration was 4.82 (4.02 to 5.70) mmol/L and TG concentration was 1.43 (1.01 to 2.04) mmol/L. After discharge, 6740 (84.4%) patients were taking statins, 4531 (56.7%) taking aspirin, 4132 (51.7%) taking clopidogrel, 4905 (61.4%) taking ACEIs/ ARBs, 4611 (57.7%) taking β-blockers and 2643 (33.1%) taking CCBs. Patients with higher Lp(a) levels were female, older, and had more hypertension, CHDs, ACS, stroke, CKD (*P* < 0.05), and higher CCIs, LDL-c and TC (Table 1).

**Table 1:** Baseline characteristics of patients

Variable	Total (*n* = 8382)	Lipoprotein(a) quartile				X^2^/R	*P*
		Quartile 1 (*n* = 2096)	Quartile 2 (*n* = 2096)	Quartile 3 (*n* = 2095)	Quartile 4 (*n* = 2095)		
Lp(a), mg/L	160.90 (80.00 to 336.41)	49.90 (35.43 to 64.98)	116.20 (96.81 to 136.80)	229.40 (190.80 to 274.20)	557.20 (422.10 to 800.10)		< 0.001*
General characteristics							
Age, years	61 (51 to 68)	59 (49 to 67)	61 (51 to 68)	62 (52 to 68)	63 (54 to 69)	0.105	< 0.001*
Male, *n* (%)	5348 (63.8)	1386 (66.1)	1365 (65.1)	1277 (61.0)	1320 (63.0)	14.415	0.002*
Local resident, *n* (%)	6504 (77.6)	1547 (73.8)	1624 (77.5)	1667 (80)	1656 (79.0)	27.097	< 0.001*
Smoking, *n* (%)	3060 (36.5)	708 (33.8)	758 (36.2)	803 (38.3)	791 (37.8)	2.579	0.461
Cardiovascular diseases							
CHDs	4338 (51.8)	985 (47.0)	1058 (50.5)	1075 (51.3)	1220 (58.2)	55.780	< 0.001*
ACS	3048 (36.4)	707 (33.7)	737 (35.2)	769 (36.7)	835 (39.9)	18.739	< 0.001*
Angina, *n* (%)	1070 (12.8)	269 (12.8)	263 (12.5)	240 (11.5)	298 (14.2)	7.329	0.061
STEMI, *n* (%)	1023 (12.2)	223 (10.6)	265 (12.6)	274 (13.1)	261 (12.5)	6.789	0.079
NSTEMI, *n* (%)	957 (11.4)	215 (10.3)	210 (10.0)	256 (12.2)	276 (13.2)	14.566	0.002*
HF, *n* (%)	69 (0.8)	15 (0.7)	11 (0.5)	19 (0.9)	24 (1.1)	5.430	0.143
AF, *n* (%)	799 (9.5)	207 (9.9)	197 (9.4)	205 (9.8)	190 (9.1)	1.007	0.800
RHDs, *n* (%)	144 (1.7)	38 (1.8)	40 (1.9)	35 (1.7)	31 (1.5)	1.294	0.730
Hypertension, *n* (%)	5117 (61.0)	1266 (60.4)	1235 (58.9)	1299 (62.0)	1317 (62.9)	8.066	0.045
Hyperlipemia, *n* (%)	1636 (19.5)	388 (18.5)	408 (19.5)	407 (19.4)	433 (20.7)	3.131	0.371
Cardiomyopathy, *n* (%)	292 (3.5)	71 (3.4)	84 (4.0)	78 (3.7)	59 (2.8)	4.902	0.179
Other diseases							
Diabetes, *n* (%)	2291 (27.3)	613 (29.2)	555 (26.5)	572 (27.3)	551 (26.3)	5.757	0.124
CKD, *n* (%)	976 (11.6)	184 (8.8)	207 (9.9)	257 (12.3)	328 (15.7)	56.669	< 0.001*
Stroke, *n* (%)	1117 (13.3)	248 (11.8)	270 (12.9)	302 (14.4)	297 (14.2)	7.873	0.049*
Other blood lipid							
LDL-c, mmol/L	3.16 (2.53 to 3.82)	2.97 (2.38 to 3.60)	3.12 (2.51 to 3.75)	3.24 (2.63 to 3.90)	3.30 (2.61 to 4.03)	0.132	< 0.001*
HDL-c, mmol/L	1.09 (0.93 to 1.30)	1.07 (0.91 to 1.28)	1.08 (0.93 to 1.27)	1.11 (0.95 to 1.31)	1.13 (0.95 to 1.33)	0.073	< 0.001*
TC, mmol/L	4.82 (4.02 to 5.70)	4.64 (3.89 to 5.53)	4.79 (4.00 to 5.61)	4.90 (4.11 to 5.76)	4.97 (4.12 to 5.94)	0.103	< 0.001*
TG, mmol/L	1.43 (1.01 to 2.04)	1.54 (1.02 to 2.45)	1.45 (1.03 to 2.05)	1.36 (0.98 to 1.89)	1.38 (0.99 to 1.92)	− 0.082	< 0.001*
CCI	3 (2 to 4)	3 (1 to 4)	3 (2 to 4)	3 (2 to 4)	3 (2 to 5)	0.102	< 0.001*
Drugs							
Statins, *n* (%)	6740 (84.4)	1565 (79.8)	1672 (83.9)	1697 (84.8)	1806 (88.9)	63.067	< 0.001*
Aspirin, *n* (%)	4531 (56.7)	1061 (54.1)	1125 (56.4)	1118 (55.9)	1227 (60.4)	17.279	0.001*
Clopidogrel, *n* (%)	4132 (51.7)	940 (48.0)	1027 (51.5)	1020 (51.0)	1145 (56.4)	29.208	< 0.001*
ACEIs/ARBs, *n* (%)	4905 (61.4)	1195 (62.4)	1244 (62.4)	1242 (62.1)	1224 (60.3)	2.504	0.475
β-blockers, *n* (%)	4611 (57.7)	1104 (56.3)	1154 (57.9)	1140 (57.0)	1213 (59.7)	5.388	0.145
CCBs, *n* (%)	2643 (33.1)	669 (34.1)	610 (30.6)	677 (33.8)	687 (33.8)	7.506	0.057

Lp(a) quartiles: Q1 ≤ 80.00 mg/L; Q2: 80.01 ~ 160.90 mg/L; Q3: 160.91 ~ 336.41 mg/L; and Q4: > 336.41 mg/L. *P < 0.05

*R* regression coefficient, ACEIs, angiotensin-converting enzyme inhibitors; *ACS* acute coronary syndrome, *AF* atrial fibrillation, *ARBs* angiotensin receptor blockers, *CCBs* calcium channel blockers, *CCI* Charlson comorbidity index, *CKD* chronic kidney disease, *CHDs* coronary heart diseases, *HDL-c* high density lipoprotein cholesterol, *HF* heart failure, *LDL-c* Low density lipoprotein cholesterol, *MI* Myocardial infarction, *NSTEMI* non-ST-segment elevation myocardial infarction, *RHDs* rheumatic heart disease, *STEMI* ST-segment elevation myocardial infarction, *TC* total cholesterol, *TG* triglyceride

During an average follow-up of 619 (320 to 1061) days, the rate of loss to follow-up was 23.7%. And 1361 (16.2%) MACEs, 124 (1.5%) myocardial infarction (MI), 418 (5.0%) unstable angina (UA), 585 (7.0%) cases of heart failure (HF), 291 (3.5%) stoke and 125 (1.5%) all-cause death were collected. The clinical outcomes are shown in Table 2.

**Table 2:** Clinical outcomes during the follow-up period

Events	Lipoprotein(a) quartile					*P*
	Total (*n* = 8382)	Quartile 1 (*n* = 2096)	Quartile 2 (*n* = 2096)	Quartile 3 (*n* = 2095)	Quartile 4 (*n* = 2095)	
MACEs, *n* (%)	1361 (16.2)	265 (12.6)	306 (17.7)	370 (17.7)	420 (20.0)	< 0.001*
MI, *n* (%)	124 (1.5)	20 (1.0)	24 (1.1)	36 (1.7)	44 (2.1)	0.008*
UA, *n* (%)	418 (5.0)	97 (4.6)	99 (4.7)	99 (4.7)	123 (5.9)	0.200
HF, *n* (%)	585 (7.0)	87 (4.2)	129 (6.2)	178 (8.5)	191 (9.1)	< 0.001*
Stroke, *n* (%)	291 (3.5)	60 (2.9)	63 (3.0)	83 (4.0)	85 (4.1)	0.062
All-cause death, *n* (%)	125 (1.5)	18 (0.9)	28 (1.3)	27 (1.3)	52 (2.5)	< 0.001*

Lp(a) quartiles: Q1 ≤ 80.00 mg/L; Q2: 80.01 ~ 160.90 mg/L; Q3: 160.91 ~ 336.41 mg/L; and Q4: > 336.41 mg/L. *P < 0.05

MACEs, major adverse cardiovascular events. *MI* myocardial infarction, *HF* heart failure, *UA* unstable angina

**Fig. 2:**
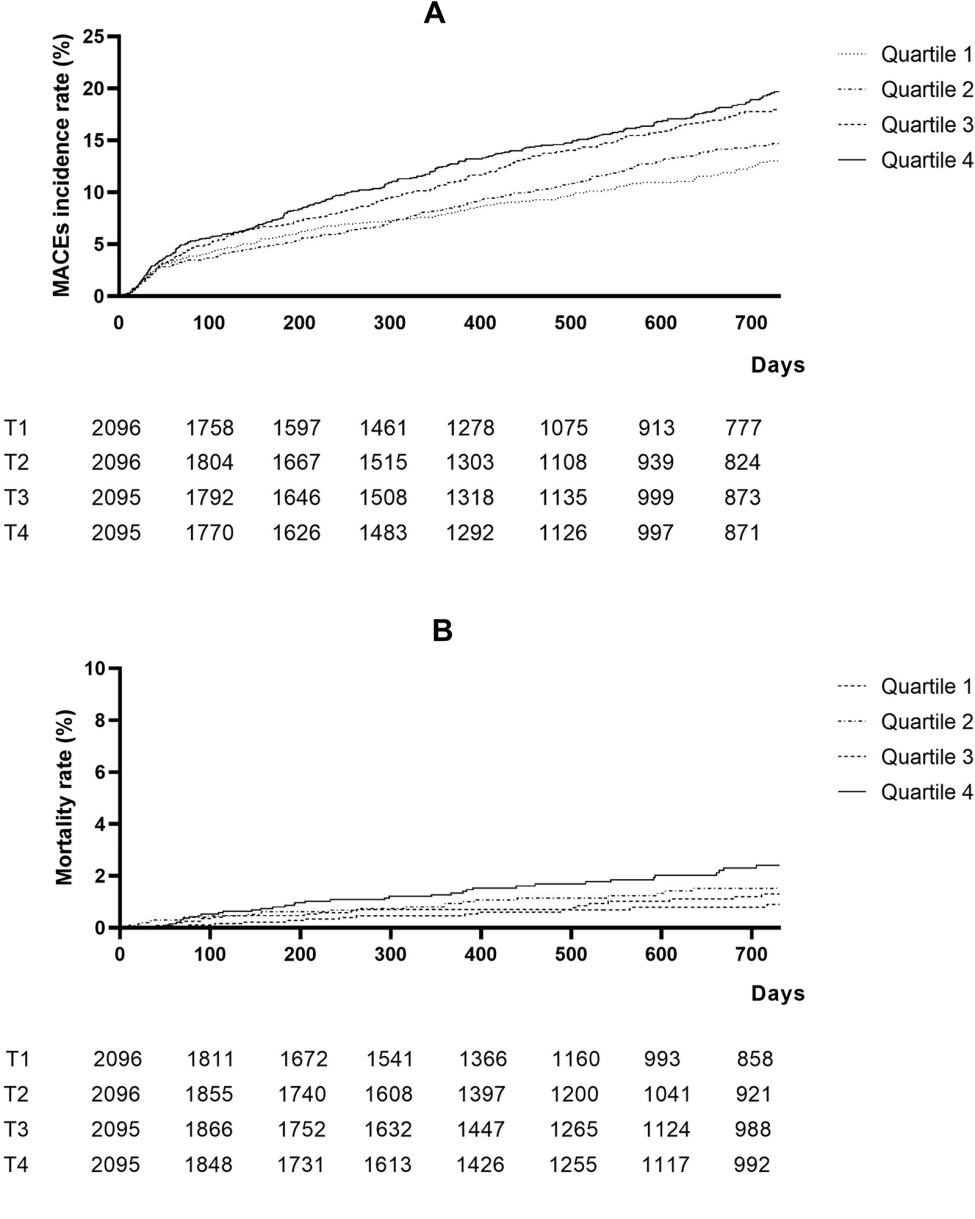
Kaplan–Meier curves according to quartiles of serum Lp(a) levels. A Major adverse cardiovascular events (MACEs); and B all-cause death. Lp(a) quartiles: Q1 ≤ 80.00 mg/L; Q2: 80.01 ~ 160.90 mg/L; Q3: 160.91 ~ 336.41 mg/L; and Q4: > 336.41 mg/L

Kaplan–Meier analysis (Fig. 2) exhibited that increased serum Lp(a) concentrations was significantly associated with the occurrence of MACEs (log-rank X^2^ = 25.767, *P* < 0.001) and all-cause deaths (log-rank X^2^ = 15.016, *P* = 0.002) in patients with cardiovascular diseases.

As shown in Table 3, the incidence of MACEs was 7.65, 8.24, 9.73 and 10.75 per 100 person-years in each Lp(a) quartile, respectively; the all-cause mortality was 0.48, 0.69, 0.64 and 1.18 per 100 person-years in each Lp(a) quartile, respectively. The incidences of both two events were found to correlate with the increase in Lp(a).

**Table 3:** Hazard ratio for MACEs and all-cause death

	Lipoprotein(a)	Lipoprotein(a) quartile			
		Quartile 1 (≤ 80.00 mg/L)	Quartile 2 (80.01 ~ 160.90 mg/L)	Quartile 3 (160.91 ~ 336.41 mg/L)	Quartile 4 (Q4: > 336.41 mg/L)
Participants, *n*		2096	2096	2095	2095
MACEs, *n*		265	306	370	420
Incidence/100 person-years		7.65	8.24	9.73	10.75
Unadjusted HR (95% CI)	1.316 (1.162 to 1.491)*	1	1.086 (0.921 to 1.280)	1.287 (1.099 to 1.507)*	1.424 (1.221 to 1.662)*
Model 1 HR (95% CI)^a^	1.189 (1.045 to 1.353)*				
Model 2 HR (95% CI)^b^		1	1.121 (0.947 to 1.328)	1.228 (1.042 to 1.448)*	1.312 (1.115 to 1.543)*
MI, *n*		20	24	36	44
Incidence/100 person-years		0.54	0.60	0.86	1.01
Unadjusted HR (95% CI)	1.615 (1.074 to 2.429)*	1	1.137 (0.647 to 1.996)	1.462 (0.860 to 2.487)	1.748 (1.048 to 2.917)*
Model 1 HR (95% CI)^a^	1.278 (0.840 to 1.947)	1			
Model 2 HR (95% CI)^b^		1	1.180 (0.657 to 2.118)	1.389 (0.795 to 2.425)	1.469 (0.853 to 2.531)
Stroke, *n*		60	63	83	85
Incidence/100 person-years		1.64	1.59	2.01	1.98
Unadjusted HR (95% CI)	1.104 (0.843 to 1.445)	1	1.958 (0.673 to 1.365)	1.207 (0.865 to 1.683)	1.177 (0.844 to 1.640)
Model 1 HR (95% CI)^a^	1.028 (0.775 to 1.363)				
Model 2 HR (95% CI)^b^		1	0.999 (0.696 to 1.435)	1.089 (0.770 to 1.541)	1.131 (0.798 to 1.601)
All-cause death, *n*		18	28	27	52
Incidence/100 person-years		0.48	0.69	0.64	1.18
Unadjusted HR (95% CI)	1.913 (1.255 to 2.916)*	1	1.446 (0.800 to 1.365)	1.335 (0.735 to 2.355)	2.464 (1.439 to 4.219)*
Model 1 HR (95% CI)^a^	1.573 (1.009 to 2.452)*				
Model 2 HR (95% CI)^b^		1	1.623 (0.868 to 3.035)	1.247 (0.660 to 1.448)	2.281 (1.270 to 4.098)*
UA, *n*		97	99	99	123
Incidence/100 person-years		2.69	2.54	2.42	2.90
Unadjusted HR (95% CI)	1.087 (0.868 to 1.360)	1	0.954 (0.721 to 1.262)	0.913 (0.690 to 1.209)	1.097 (0.840 to 1.433)
Model 1 HR (95% CI)^a^	0.977 (0.776 to 1.231)				
Model 2 HR (95% CI)^b^		1	0.922 (0.691 to 1.230)	0.907 (0.679 to 1.212)	0.979 (0.742 to 1.293)
HF, *n*		87	129	178	191
Incidence/100 person-years		2.39	3.31	4.47	4.61
Unadjusted HR (95% CI)	1.673 (1.383 to 2.025)*	1	1.404 (1.071 to 1.840)*	1.908 (1.478 to 2.463)*	1.995 (1.549 to 2.569)*
Model 1 HR (95% CI)^a^	1.501 (1.231 to 1.831)*				
Model 2 HR (95% CI)^b^		1	1.549 (1.172 to 2.047)*	1.754 (1.343 to 2.292)*	1.885 (1.441 to 2.466)*

Lp(a) quartiles: Q1 ≤ 80.00 mg/L; Q2: 80.01 ~ 160.90 mg/L; Q3: 160.91 ~ 336.41 mg/L; and Q4: > 336.41 mg/L. * P < 0.05

Model 1 and Model 2 were adjusted by age, sex, hypertension, history of CHDs, CKD, stroke, CCI, statins, HDL-c, TC, and LDL-c. Model 1^a^ used Lp(a) as a continuous variable; Model 2^b^ used Lp(a) as a categorical variable. *MACEs* major adverse cardiovascular events, *MI* myocardial infarction, *UA* unstable angina, *HF* heart failure

When Lp(a) was treated as a continuous variable, the incidence of MACEs would increased by 31.6% (HR: 1.316, [95% CI: 1.162 to 1.491], *P* < 0.001) for every 1 mg/L increase in Lp(a). For all-cause deaths, mortality was increased by 91.3% for every 1 mg/L increase in Lp(a) (HR: 1.913, [95% CI: 1.255 to 2.916], *P* = 0.003). After adjusting the age, sex, hypertension, history of CHDs, CKD, stroke, CCI, statins, HDL-c, TC, and LDL-c, the trends had not changed.

According to Table 3, when Lp(a) was used as a categorical variable, compared with group Q1, the unadjusted HR of group Q2, Q3 and Q4 in MACEs was 1.086 (95% CI: 0.921 to1.280, *P* = 0.325) and 1.287 (95% CI:1.099 to1.507, *P* = 0.002) and 1.424 (95% CI: 1.221 to 1.662, *P* < 0.001), respectively. Similarly, compared with group Q1, the unadjusted HR for groups Q2, Q3 and Q4 in all-cause death was 1.446 (95% CI: 0.800 to 1.365, *P* = 0.223) and 1.335 (95% CI:0.735 to 2.355, *P* = 0.343) and 2.464 (95% CI: 1.439 to 4.219, *P* = 0.001), respectively. After adjusting the age, sex, hypertension, history of CHDs, CKD, stroke, CCI, statins, HDL-c, TC, and LDL-c, the trends were similar to before, indicated that the risk of MACEs was increased when Lp(a) > 160.90 mg/L, and the risk of all-cause death was increased when Lp(a) > 336.41 mg/L.

In addition, after adjusting the age, sex, hypertension, history of CHDs, CKD, stroke, CCI, statins, HDL-c, TC, and LDL-c, multivariate COX regression analysis suggested that male, CKD, stroke, CCI and TC were independent risk factors for MACEs. And there was no significant correlations between MACEs and age, smoking, place of residence, hypertension and CHD. For all-cause death, hypertension, CKD, stroke, CCI were independent risk factors. And statins were effective protective factors in both two events. After adding an interaction term between Lp(a) and statin into the Cox model for the overall population, the result suggested that there was no interaction term between Lp(a) and statins (HR: 0.824, [95% CI: 0.590 to 1.152], *P* = 0.258) (Tables 4, 5).

**Table 4:** Cox regression analysis about MACEs in patients with cardiovascular diseases

Variables	Univariable		Model 1^a^		Model 2^b^	
	HR (CI 95%)	*P* value	HR (CI 95%)	*P* value	HR (CI 95%)	*P* value
Gender	1.424 (1.267 to 1.600)	< 0.001*	1.369 (1.203 to 1.556)	< 0.001*	1.371 (1.206 to 1.559)	< 0.001*
Age	1.025 (1.020 to 1.031)	< 0.001*	1.002 (0.995 to 1.008)	0.648	1.001 (0.995 to 1.008)	0.697
Smoking	0.927 (0.773 to 1.112)	0.415				
Local resident	1.057 (0.920 to 1.214)	0.434				
Lp(a)	1.133 (1.080 to 1.189)	< 0.001*	1.189 (1.045 to 1.353)	0.008*		
Q2 vs. Q1	1.086 (0.921 to 1.280)	0.325			1.121 (0.947 to 1.328)	0.186
Q3 vs. Q1	1.287 (1.099 to 1.507)	0.002*			1.228 (1.042 to 1.448)	0.014*
Q4 vs. Q1	1.424 (1.221 to 1.662)	< 0.001*			1.312 (1.115 to 1.543)	0.001*
Hypertension	1.126 (1.007 to 1.258)	0.037*	0.911 (0.809 to 1.025)	0.122	0.914 (0.812 to 1.029)	0.136
Hyperlipemia	0.934 (0.814 to 1.071)	0.330				
CHDs	1.319 (1.184 to 1.469)	< 0.001*	1.037 (0.913 to 1.179)	0.574	1.036 (0.912 to 1.178)	0.586
MI	1.035 (0.919 to 1.165)	0.575				
CKD	2.584 (2.283 to 2.924)	< 0.001*	1.778 (1.552 to 2.036)	< 0.001*	1.771 (1.547 to 2.028)	< 0.001*
Stroke	1.605 (1.403 to 1.837)	< 0.001*	1.325 (1.113 to 1.577)	0.002*	1.325 (1.113 to 1.578)	0.002*
Statins	0.809 (0.703 to 0.933)	0.003*	0.616 (0.526 to 0.721)	< 0.001*	0.616 (0.526 to 0.721)	< 0.001*
Aspirin	1.029 (0.921 to 1.148)	0.616				
Clopidogrel	1.061 (0.951 to 1.183)	0.291				
CCI	1.247 (1.221 to 1.274)	< 0.001*	1.244 (1.202 to 1.287)	< 0.001*	1.244 (1.202 to 1.286)	< 0.001*
LDL-c	0.940 (0.890 to 0.994)	0.029*	0.874 (0.767 to 0.995)	0.042*	0.867 (0.762 to 0.987)	0.031*
HDL to c	0.575 (0.473 to 0.698)	< 0.001*	0.778 (0.624 to 0.971)	0.026*	0.774 (0.621 to 0.966)	0.023*
TC	0.950 (0.911 to 0.990)	0.016*	1.131 (1.024 to 1.250)	0.015*	1.135 (1.028 to 1.253)	0.012*
TG	0.985 (0.947 to 1.024)	0.436				

Lp(a) quartiles: Q1 ≤ 80.00 mg/L; Q2: 80.01 ~ 160.90 mg/L; Q3: 160.91 ~ 336.41 mg/L; and Q4: > 336.41 mg/L. *P < 0.05

Model 1^a^ used Lp(a) as a continuous variable; Model 2^b^ used Lp(a) as a categorical variable. *CCI* Charlson comorbidity index, *CHDs* coronary heart diseases, *CKD* chronic kidney disease, *HDL-c* high density lipoprotein cholesterol, *LDL-c* Low density lipoprotein cholesterol, *MI* myocardial infarction, *TC* total cholesterol, *TG* triglyceride

**Table 5:** Cox regression analysis about all-cause death in patients

Variables	Univariable		Model 1^a^		Model 2^b^	
	HR (CI 95%)	*P* value	HR (CI 95%)	*P* value	HR (CI 95%)	*P* value
Male	1.165 (0.802 to 1.693)	0.423	1.049 (0.697 to 1.580)	0.817	1.034 (0.686 to 1.558)	0.873
Age, years	1.037 (1.018 to 1.057)	< 0.001*	0.992 (0.969 to 1.015)	0.477	0.991 (0.968 to 1.014)	0.451
Smoking	0.898 (0.622 to 1.297)	0.567				
Local resident	1.921 (1.081 to 3.416)	0.026*				
Lp(a)	1.913 (1.255 to 2.916)	0.003*	1.573 (1.009 to 2.452)	0.046		
Q2 vs Q1	1.446 (0.800 to 2.614)	0.223			1.623 (0.868 to 3.035)	0.130
Q3 vs Q1	1.335 (0.735 to 2.424)	0.343			1.247 (0.660 to 1.448)	0.496
Q4 vs Q1	2.464 (1.439 to 4.219)	0.001*			2.281 (1.270 to 4.098)	0.006*
Hypertension	0.801 (0.562 to 1.141)	0.219	2.040 (1.386 to 3.003)	< 0.001*	2.008 (1.364 to 2.957)	< 0.001*
Hyperlipemia	0.940 (0.598 to 1.477)	0.788				
CHDs	0.794 (0.558 to 1.129)	0.199	1.786 1.155 to 2.761)	0.403	1.828 (1.182 to 2.827)	0.007*
MI	1.138 (0.775 to 1.671)	0.509				
CKD	4.548 (3.171 to 6.524)	< 0.001*	2.725 (1.802 to 4.121)	< 0.001*	2.675 (1.769 to 4.045)	< 0.001*
Stroke	1.932 (1.275 to 2.926)	0.002*	2.124(1.195 to 3.775)	< 0.001*	2.089 (1.172 to 3.725)	0.012*
Statins	0.457 (0.304 to 0.687)	< 0.001*	0.471 (0.280 to 0.750)	0.002*	0.463 (0.291 to 0.735)	0.001*
Aspirin	0.564 (0.389 to 0.818)	0.003*				
Clopidogrel	0.834 (0.580 to 1.201)	0.329				
CCI	1.378 (1.295 to 1.467)	< 0.001*	1.496 (1.346 to 1.662)	< 0.001*	1.496 (1.346 to 1.663)	< 0.001*
LDL-c	0.867 (0.720 to 1.044)	0.131	0.849 (0.549 to 1.313)	0.461	0.845 (0.550 to 1.300)	0.444
HDL-c	0.276 (0.138 to 0.500)	< 0.001*	0.505 (0.238 to 1.070)	0.075	0.492 (0.232 to 1.043)	0.064
TC	0.865 (0.749 to 0.998)	0.048*	1.157 (0.829 to 1.614)	0.391*	1.152 (0.829 to 1.601)	0.400*
TG	1.010 (0.899 to 1.135)	0.865				

Lp(a) quartiles: Q1 ≤ 80.00 mg/L; Q2: 80.01 ~ 160.90 mg/L; Q3: 160.91 ~ 336.41 mg/L; and Q4: > 336.41 mg/L. *P < 0.05

Model 1^a^ used Lp(a) as a continuous variable; Model 2^b^ used Lp(a) as a categorical variable. *CCI* Charlson comorbidity index, *CHDs* coronary heart diseases, *CKD* chronic kidney disease, *HDL-c* high density lipoprotein cholesterol, *LDL-c* low density lipoprotein cholesterol, *MI* myocardial infarction, *TC* total cholesterol, *TG* triglyceride

**Fig. 3:**
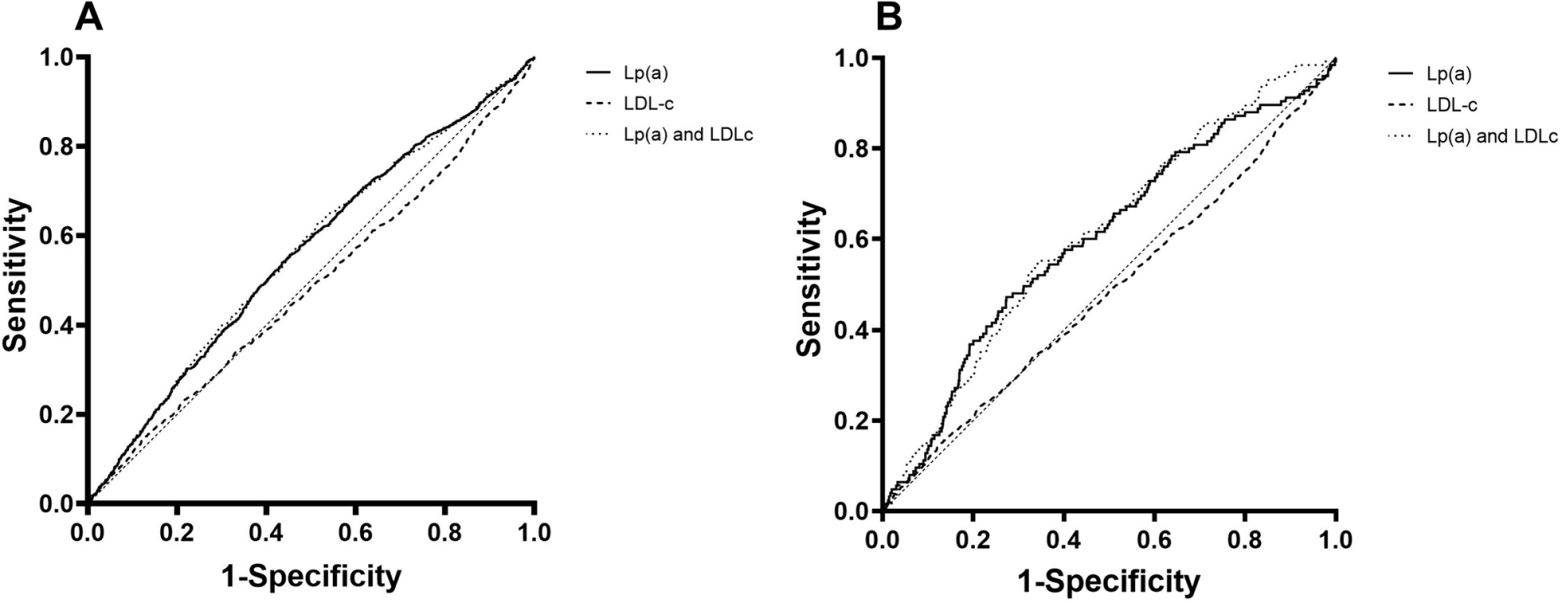
The receiver operating characteristic (ROC) curve. **A** MACEs, **B** all-cause death

The ROC curves are shown in Fig. 2. For MACEs, the AUC of serum Lp(a) concentrations [0.559, (95% CI: 0.542 to 0.575), *P* < 0.001] was greater than LDL-c [0.485, (95% CI: 0.467 to 0.502), *P* = 0.073]. When combined the Lp(a) with LDL-c, the combination AUC of Lp(a) and LDL-c [0.556, (95% CI: 0.539 to 0.574), *P* < 0.001] did not show any advantages than Lp(a) (Fig. 3A). For all-cause death in Fig. 3B, the AUC of serum Lp(a) concentrations 0.600 [0.600, (95% CI: 0.549 to 0.651), *P* < 0.001] was greater than LDL-c [0.462, (95% CI: 0.142 to 0.406), *P* = 0.142]. The combination AUC of Lp(a) and LDL-c [0.611, (95% CI: 0.563 to 0.658), *P* < 0.001] had shown remarkable advantages than Lp(a) and LDL-c alone.

## Discussion

Lipoprotein(a), one of the components of blood lipids, was described as a cardiovascular risk factor in many studies [16]. Lp(a) can increase the risk of thrombosis by preventing the binding of plasminogen to the platelets surface and endothelial cells [17, 18]. Meanwhile, high serum Lp(a) concentrations can accelerate atherogenesis [19]. In this study, we retrospectively examined the association between serum Lp(a) levels and the risk of MACEs/all-cause death in hospitalized patients with cardiovascular diseases, and found high Lp(a) levels were closely related with MACEs and all-cause death.

Analysis indicated that there might be a threshold between the Lp(a) and major cardiovascular events. When Lp(a) > 160.90 mg/L, there was a dose–response effect for MACEs. And the risk of all-cause death was increased when Lp(a) > 336.41 mg/L. The threshold differed from many previous studies. Because of the lack of standardization of the testing methods [16], it is complicated to standardize the control objective of Lp(a). Kostner et al. [20] conducted a case–control study on 76 patients with previous acute myocardial infarction, and indicated that Lp(a) represented an independent risk factor for MI, when Lp(a) concentrations above 300 mg/L. Suk et al. [21] found women whose Lp(a) concentrations above 440 mg/L were more likely to develop cardiovascular events. Kamstrup et al. [22] demonstrated that an extreme serum Lp(a) level resulted in a 3- to fourfold increase in MI in patients. And found a dose–response relationship between them, when the Lp(a) concentrations reached a high level (> 95%). A review from the Beijing Heart Society and Expert Committee [23], which compared several studies involving different regions and ethnicity, found that Lp(a) ≥ 300 mg/L is an independent predictor of CAD and ischemic stroke. These findings suggested that Lp(a) levels might be influenced by region and lifestyle. Meanwhile, our research indicated that regardless of the types of cardiovascular diseases (including valvular heart diseases, cardiomyopathy, etc.), aggressively to reduce Lp(a) concentration may contribute to reduce the incidence of MACEs. However, some studies like Matti Jauhiainen et al. [24] did not support the close relationship between Lp(a) and coronary diseases, this might be related to the sources and storage method of samples.

As a cholesterol-rich lipoprotein [25, 26], the contribution of Lp(a) to serum cholesterol should be taken into account. In the baseline table, patients with serum Lp(a) concentrations appeared to have higher serum cholesterol levels, this indicated that high serum Lp(a) would lead to an increase of cholesterol. Len et al. [27] found that patients with serum cholesterol levels above 6.5 mmol/L and Lp(a) levels above 200 mg/L were more likely to suffer an acute myocardial infarction. Thus, despite Lp(a) is a relatively modest coronary factor, the effect on cholesterol should be taken into concern. Our analysis showed that statin therapy had a significant protective effect to reduce the risk of MACEs/ all-cause death. Although the mechanism is still not fully understood, statins may improve outcomes by reducing blood cholesterol. After adjusting for medications and other factors, including statins, Lp(a) still remained an independent risk factor for MACEs. And the study did not find a interaction between statins and Lp(a), suggesting that early using of statins can improve the outcomes, but patients with high Lp(a) levels still need attentions, and Lp(a) may become a new target for the prevention of atherosclerotic diseases. Niacin, a broad-spectrum lipid-regulating drug, was regarded as the only drug that can specifically reduce the circulating levels of Lp(a) by up to 30% [28]. O’Donoghue et al. [29] demonstrated that olpasiran, a small interfering RNA, reduced serum Lp(a) concentrations in a dose-dependent manner. Despite the effect of cardiovascular adverse events still needs to be further explored, it may provide directions for future lipid regulation strategies.

It should be noted that we found that some blood lipids (especially LDL and TG, which have been demonstrated have a significant effect on cardiovascular diseases [30–32]) showed a protective effect in outcomes. This might be for two reasons. First, most patients with hyperlipidemia started medication (especially statins) early, so a rising blood lipid might represent more aggressive treatments. Second, it might be due to the poor nutritional status of patients with chronic and severe cardiovascular diseases. So, higher blood lipid indicated better nutritional status, and those patients with elevated lipids might have a better outcome than those who had a low blood lipid. But in these two situations, the higher Lp(a) concentrations still showed a close association with MACEs and all-cause death.

There are several inherent limitations to the study: (1) in order to more comprehensively explore the correlation between the prognosis of patients with cardiovascular diseases and Lp(a), our study included a variety of cardiovascular diseases, and did not screen past treatment experience. Therefore, this would have an impact on the prognosis, disease progression and biochemical indicators of patients to some extent; (2) therapeutic strategies, such as drug optimization and interventional therapy, were not evaluated in this study, which might affect the clinical outcomes of patients admitted with myocardial infarction; (3) all patients in this study strictly followed the randomization principle and established relevant exclusion criteria. However, the data collection was difficult to cover patients who died before hospital or asymptomatic patients, which might produce bias. The follow-up control and subgroup analysis should be added to strengthen the conviction of the conclusion; (4) most of the population of our study were from single medical center, because serum Lp(a) concentrations were closely related to gene expression [33] and race [34], our conclusion was difficult to represent the situation of patients in other parts of China. Thus, in order to further prove the validity of the results, we need a multi-center study to evaluate the connection between serum Lp(a) and MACEs, all-cause death in populations from different regions and ethnicities.

## Conclusion

In addition to traditional lipid indicators, higher Lp(a) exhibited higher risks of adverse cardiovascular events and death, indicating worse prognosis. Lp(a) may be a new target for the prevention of atherosclerotic diseases.

## Data Availability

The original contributions presented in the study are included in the article/supplementary material, further inquiries can be directed to the corresponding author.
